# Enhancing hair regrowth using rapamycin-primed mesenchymal stem cell-derived exosomes

**DOI:** 10.7150/thno.107659

**Published:** 2025-06-09

**Authors:** Manju Shrestha, Tiep Tien Nguyen, Rabyya Kausar, Dinesh Chaudhary, Ines Ferdiana Puspitasari, Hu-Lin Jiang, Hyung-Sik Kim, Simmyung Yook, Jee-Heon Jeong

**Affiliations:** 1 Department of Precision Medicine, School of Medicine, Sungkyunkwan University, Suwon 16419, Republic of Korea.; 2 State Key Laboratory of Natural Medicines, China Pharmaceutical University, Nanjing, 210009, China.; 3 Jiangsu Key Laboratory of Druggability of Biopharmaceuticals, China Pharmaceutical University, Nanjing, 210009, China.; 4 Jiangsu Key Laboratory of Drug Discovery for Metabolic Diseases, China Pharmaceutical University, Nanjing, 210009, China.; 5 NMPA Key Laboratory for Research and Evaluation of Pharmaceutical Preparations and Excipients, China Pharmaceutical University, Nanjing, 210009, China.; 6 Department of Oral Biochemistry, Dental and Life Science Institute, Pusan National University, Yangsan, 50612, Republic of Korea.; 7 Department of Life Science in Dentistry, School of Dentistry, Pusan National University, Yangsan, 50612, Republic of Korea.; 8 Education and Research Team for Life Science on Dentistry, Pusan National University, Yangsan, 50612, Republic of Korea.; 9 School of Pharmacy, Sungkyunkwan University, Suwon, Gyeonggi, 16419, Republic of Korea.; 10 Department of Biopharmaceutical Convergence, Sungkyunkwan University, Suwon, 16419, Republic of Korea.

**Keywords:** mesenchymal stem cell, exosome, rapamycin, hair regrowth, Wnt/β-catenin signaling

## Abstract

**Rationale:** Hair loss affects millions globally, with limited effective treatments available and significant psychological impacts. Mesenchymal stem cells (MSCs) and MSC-derived exosomes hold therapeutic potential by modulating cellular communication, reducing inflammation, and supporting hair follicular regeneration. Rapamycin, a mechanistic target of rapamycin (mTOR) inhibitor, enhances MSC therapeutic potential by promoting the release of growth factors and signaling molecules. Thus, this study explores the benefit of priming effect of rapamycin on enhancing the function of MSC-derived exosomes to promote hair regrowth in a depilation-induced murine model.

**Methods:** MSCs were primed with rapamycin, and exosomes were extracted from the MSC-conditioned media using ultrafiltration and poly (ethylene glycol) (PEG) precipitation. Dermal fibroblasts were treated with several doses of exosomes to evaluate the *in vitro* effect of rapamycin-primed MSC-derived exosomes (REXO). The depilated mice were administered exosomes via intradermal route and the hair regrowth was monitored for 15 days, followed by gene expression analysis and histological examination.

**Results:** Dermal fibroblasts treated with REXO showed a higher proliferation rate and an increase in genes related to Wnt/β-catenin signaling, autophagy, and growth factors compared to non-primed MSC-derived exosomes (CEXO). *In vivo* REXO therapy via intradermal injection to the depilated areas in mice enhanced hair follicle development, hair density, and hair activation markers compared with the control and naive exosome treatments.

**Conclusion:** REXO therapy effectively enhances hair regrowth thus this approach could offer a clinically effective therapy for hair loss treatment.

## Introduction

Hair loss is a widespread and distressing condition that affects millions worldwide, often leading to significant psychological impact and a reduced quality of life 1-3. Traditional treatments, such as minoxidil and finasteride, offer limited efficacy and can have undesirable side effects such as dermatitis, scalp irritation, and sexual dysfunction, highlighting the need for more innovative and effective therapeutic strategies 4. While hair transplantation can be effective, it is invasive and costly, with results that may vary depending on the technique and the surgeon's expertise 5. Although platelet-enriched plasma therapy and low-intensity laser therapy are less invasive alternatives, they provide variable results and often require multiple treatments to maintain efficacy 6. These limitations underscore the ongoing need for new, more effective treatments, as the search for less invasive and more reliable therapies continues.

Stem cell therapies, particularly those involving mesenchymal stem cells (MSCs), have shown promise in promoting hair growth. Stem cells initiate the shift of the hair cycle from the telogen phase (resting) to the anagen phase (growing) 7. MSCs have been shown to maintain the hair follicle immune privilege by their immunomodulatory functions and enhance hair follicle regeneration 8. Previous studies have also demonstrated that MSCs could increase hair density 9, 10 and hair diameter 11. These findings highlight the potential of MSCs to promote hair regeneration and offer as a promising avenue for treating hair loss. However, there is a risk of immune rejection and tumorigenicity, which limits the clinical use of MSCs for hair growth 12. Furthermore, autologous MSCs require more invasive procedures for cell harvesting and administration, and there are concerns regarding their large-scale production, long-term storage, and viability 13, 14. Additionally, the precise mechanisms through which MSCs promote hair growth are not fully understood, thereby complicating the optimization and standardization of treatment protocols 15, 16.

To address the limitations associated with MSCs, exosome-based therapies have emerged as a breakthrough solution 17. MSCs release a broad array of paracrine factors, which contribute for up to 80% of their therapeutic efficacy 18. Exosomes, small extracellular vesicles (EVs, 50-200 nm), are among the paracrine factors released by MSCs and are considered as key mediators of intercellular communication by delivering proteins, mRNAs, and miRNAs to surrounding cells 19. Exosomes possess several advantages over MSCs, including longer storage time, easier transportation 20, and the ability to deliver therapeutic molecules more effectively by directly fusing to target cells 21. In addition, factors such as concentration, dosage, administration route, and timing can be easily controlled. Unlike MSC therapy, MSC-exosomes eliminate the risk of aneuploidy and immune rejection 18, 22. Consequently, the use of MSC-derived exosomes as cell-free therapy has recently emerged as a potential therapeutic approach for hair regrowth. Previous studies have also demonstrated that these exosomes can enhance hair follicle development and stimulate hair regrowth due to their ability to modulate the local cellular environment and promote angiogenesis, cellular proliferation, and differentiation 23. Recent studies have further confirmed that MSC-derived exosomes enhance hair follicle activation and facilitate the transition to the anagen phase through Wnt/β-catenin and RAS/ERK pathways, underscoring their promising role in hair regeneration 24, 25. In addition, MSC-exosomes can promote cutaneous wound healing 26, tissue repair 27, and inflammatory response regulation 28, and they have been shown to enhance the proliferative and differentiative potential of dermal papilla fibroblasts, which have a major role in the development and cycling of hair follicles 29.

Rapamycin, a well-known immunosuppressant and mechanistic target of rapamycin (mTOR) pathway inhibitor, has been implicated in enhancing tissue regeneration 30. mTOR inhibition by rapamycin promotes autophagy, improves cell survival under stress conditions, and enhances immunomodulatory functions 31. MSCs primed with rapamycin has frequently demonstrated enhanced therapeutic efficacy in various diseases 32, 33. Rapamycin priming also promotes the release of growth factors and signaling molecules that support hair follicle regeneration and reduce inflammation, which is often associated with hair loss disorders 34, 35. Moreover, autophagy has been shown to induce hair follicle stem cell activation and regeneration by modulating glycolysis, providing additional mechanistic support for the potential of rapamycin-primed exosomes in hair regrowth applications 36.

**Figure 1:**
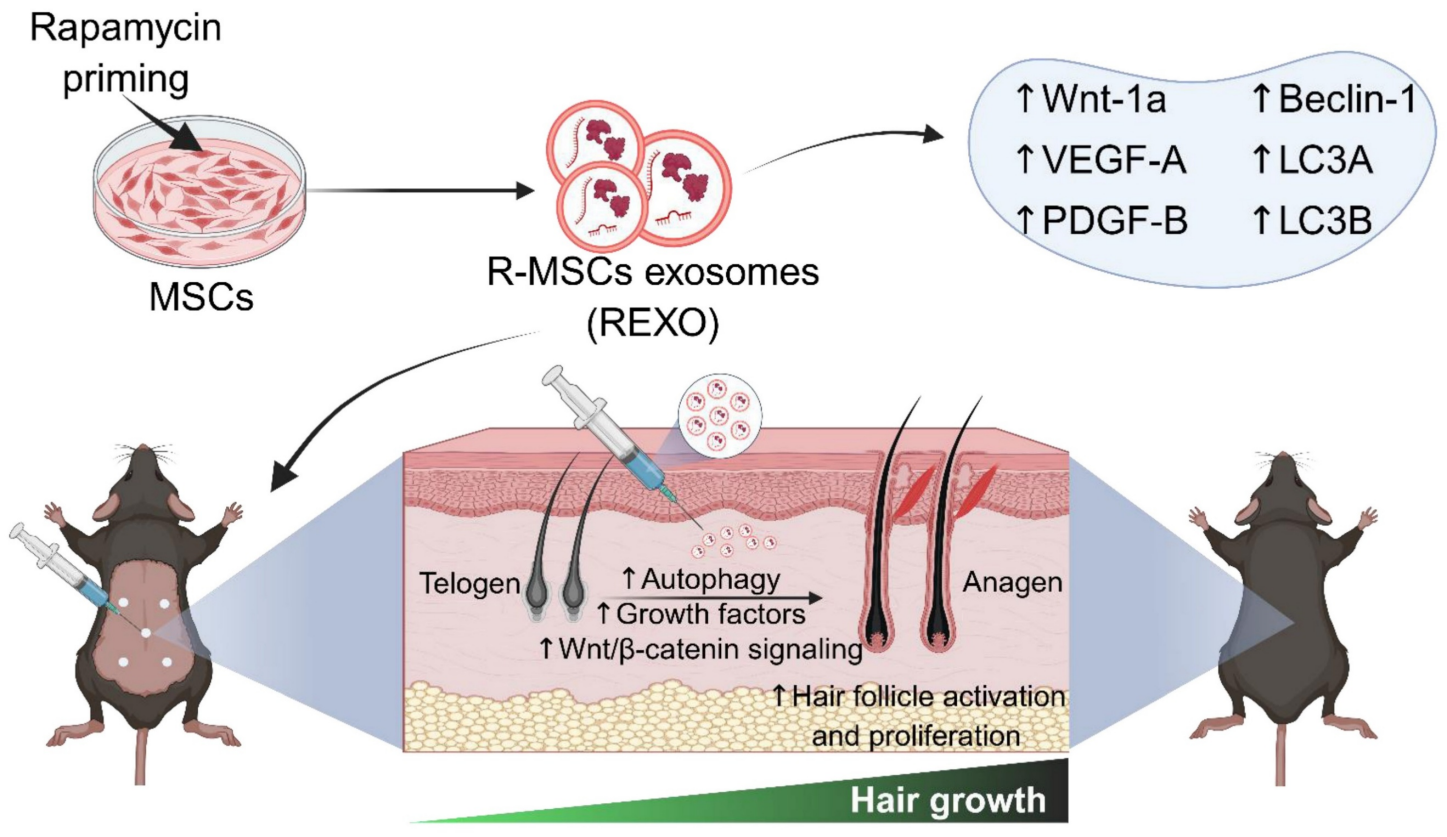
** Engineering of MSCs with rapamycin (R) to enhance exosome (EXO) release and function, promoting hair regrowth in an experimental mouse model.** REXO therapy exhibited its therapeutic effects via modulation of Wnt/β-catenin signaling and autophagy, thereby promoting hair follicle activation and effective hair regrowth. (The figure was designed with Biorender.com).

As rapamycin is a well-established inducer of autophagy through mTOR inhibition, and MSC-derived exosomes are known to deliver bioactive molecules that promote tissue regeneration, we hypothesized that priming MSCs with rapamycin could selectively enhance the therapeutic cargo of their exosomes, particularly factors involved in autophagy, angiogenesis and Wnt/β-catenin signaling, which are crucial factors for hair growth. This synergistic approach is expected to amplify the regenerative capacity of exosomes, and offer a novel, cell-free therapeutic strategy for hair loss, thereby improving dermal fibroblast activation, hair follicle cycling, and overall hair regrowth (Figure 1).

Based on this rationale, our study aimed to evaluate the regenerative potential of rapamycin-primed MSC-derived exosomes (REXO) in promoting hair regrowth in a depilated C57BL/6 mouse model. We envision that REXO therapy could offer a clinically effective therapeutic approach for hair loss treatment.

## Materials and Methods

### Isolation and characterization of mouse adipose MSCs

The isolation of MSCs was initiated by harvesting subcutaneous adipose tissues from 8-10-week-old C57BL/6 mice (male, Orient Bio, Seongnam, Gyeonggi-do, Republic of Korea). In brief, the euthanized mice were disinfected by dipping in 70% ethanol. Subcutaneous adipose tissues were exposed and collected after removing the inguinal lymph nodes. The tissues were then minced and incubated with 0.1% collagenase P solution (Sigma-Aldrich, MA, USA) at 37 °C for 30 min. Tissues were subsequently neutralized by Dulbecco's Modified Eagle Medium (DMEM) media (Bylabs, Hanam, Gyeonggi-do, Republic of Korea) comprising 10% fetal bovine serum (FBS, Gibco, USA) and 1% penicillin/streptomycin antibiotics (P/S) 100X (Bylabs, Hanam, Gyeonggi-do, Republic of Korea) and centrifuged at 500 × g for 5 min. The cells were further purified via a 40-μm cell filter to any remaining fat cells, and subsequently cultured at 37 °C. The unattached cells and debris were rinsed with phosphate-buffered saline (PBS) the next day, and the culture was continued until 90% confluence. In our experiments, we only used MSCs from passages 3-5.

The isolated MSCs were first characterized based on their potential to differentiate into various lineages. MSCs were stimulated to differentiate into osteogenic, adipogenic, and chondrogenic cell types using specific differentiation media 37. For initiation of osteogenic differentiation, the cells were cultured with osteogenic induction media containing dexamethasone, β-glycerophosphate, and ascorbate for 7 days. Furthermore, adipogenic differentiation was induced using media containing insulin, dexamethasone, and indomethacin for 21 days. For chondrogenic differentiation, cell pellets were prepared by embedding the MSCs in 2% methylcellulose, facilitating spheroid formation. The spheroids were cultured in chondrogenic differentiation media supplemented with TGF-β3 for 21 days. After incubation for the appropriate number of days, differentiation was confirmed through staining methods such as alkaline phosphatase, Oil Red O, and Alcian blue for osteogenesis, adipogenesis, and chondrogenesis, respectively. In addition, MSCs were assessed for surface markers through flow cytometry. The cells were labeled with fluorochrome-conjugated antibodies against CD90, CD44, CD29 and Sca-1 as positive markers, and CD11b and CD45 as negative markers.

### Isolation and characterization of the exosomes

First, MSCs (1 × 10^6^ cells) were cultured in complete DMEM media having 1% P/S and 10% FBS for 24 h at 37 °C with 5% CO_2._ Then, the cells were washed with PBS on the next day and cultured with FBS-free DMEM containing 1% P/S with or without the presence of 0.001 mM rapamycin for the next 24 h. The collected cell culture conditioned medium was then sequentially centrifuged at 300 × g for 10 min, then at 2000 × g for 10 min, followed by 10,000 × g for 30 min to discard dead cells, cell debris, and larger vesicles, respectively. The final supernatant was transferred via a 0.22-µm strainer. In this study, exosomes were isolated through a method involving both ultrafiltration and poly (ethylene glycol) (PEG) precipitation. Briefly, the filtered supernatant was transferred to ultrafiltration tubes (100 kDa MWCO; Sartorius, Germany) and centrifuged at 3000 × g and 4 °C. This process was used to eliminate a large portion of soluble protein contamination and to obtain a 25-fold concentrate. Next, the concentrate was incubated with the same volume of 1000 mM sodium chloride solution containing 16% PEG 8000 (Sigma-Aldrich, USA) while shaking at 4 ℃ overnight. Subsequently, the precipitates were settled by centrifugation at 3000 × g at 4 °C for 30 min, and resuspended in PBS to obtain CEXO (exosomes without rapamycin priming) and REXO (with rapamycin priming) for downstream analysis, or they were preserved at -80 °C.

The EXO size distribution, concentration, and purity were quantified by nanoparticle tracking analysis (NTA) (Malvern NanoSight NS300, UK). The EXO samples were first diluted in PBS to a suitable concentration to avoid particle aggregation and multiple scattering events. Approximately 1 mL of the diluted exosome solution was loaded into the NTA instrument. Particle concentration (particles/mL) and size distribution were recorded, with triplicate readings averaged for each sample. Purity was calculated by dividing the particle concentration by the respective total protein concentration (µg/mL), as measured by the Bicinchoninic acid (BCA) assay.

The EXO morphology was assessed using transmission electron microscopy (TEM). The samples were prepared following the method described earlier, with slight modifications 38. In brief, the samples were fixed using 2% paraformaldehyde (PFA) and transferred on the carbon-coated copper grids. After 20 min of incubation, the grids were rinsed with PBS and counterstained using 2% uranyl acetate for 1 min. The grids were allowed to air-dry and visualized under a transmission electron microscope at appropriate magnification, to capture image confirming the EXO structure and size.

Exosomal markers were evaluated by Western blot analysis. First, RIPA buffer was used for the lysis of the EXO formulations, and the total concentration of protein was measured by the Pierce^TM^ BCA protein assay kit (Thermo Scientific, USA). 20 µg of protein were run on a 12% SDS-PAGE gel and transferred to a polyvinylidene difluoride (PVDF) membrane (Immobilon-P; Merck Millipore, USA). One hour membrane blocking was done with 5% bovine serum albumin (BSA; Thermo Fisher Scientific, USA) prepared in tris-buffer consisting of 0.05% Tween 20 at room temperature (RT) and treated with primary antibodies targeting either rat anti-mouse CD9 (1:1000, BioLegend, USA), mouse anti-mouse CD81 (1:1000, BioLegend, USA), rat anti-mouse CD63 (1:1000, BioLegend, USA), rabbit anti-mouse Rab27A (1:1000, Cell Signaling Technology, USA), rabbit anti-mouse HSP70 (1:1000, Cell Signaling Technology, USA), or rat anti-mouse calnexin (1:1000, BioLegend, USA) at 4 °C overnight. After washing, the membranes were left to incubate at RT for 1 h along with goat anti-mouse IgG (H + L)-HRP (1:10000; Invitrogen, USA) for the CD81 marker, goat anti-rabbit IgG (H + L) HRP (1:10000; Invitrogen, USA) for Rab27A and HSP70,and with goat anti-rat IgG (H + L)-HRP (1:10000; Thermo Fisher Scientific, USA) for the remaining markers. Lastly, the signals were developed with Amersham ECL^TM^ Prime Western Blotting Detection Reagents (Cytiva, USA). Band intensities were examined by Fujifilm LAS-3000 Imager (Fujifilm, Tokyo, Japan).

### Isolation and treatment of dermal fibroblasts with exosomes

Dermal fibroblasts were isolated from the dorsal skin of 6-week-old male C57BL/6 mice (Orient bio, Seongnam, Gyeonggi-do, Republic of Korea), as previously described with slight modifications 39-41. Briefly, the mice were euthanized by CO_2_ inhalation, followed by dorsal hair removal and gentle sterilization with 70% ethanol. The skin was excised and incubated with 0.1% dispase (STEMCELL Technologies, Canada) at 37 °C for 1.5 h to separate the epidermis and dermis. After incubation, the epidermal layer was gently peeled off using sterile forceps. The dermal layer was finely minced into small fragments and digested in 0.1% collagenase type P solution (Sigma-Aldrich, MA, USA) at 37 °C for 45 min. The cells were collected by centrifugation at 500g for 5 min and the resulting pellet was resuspended in DMEM (Bylabs, Hanam, Gyeonggi-do, Republic of Korea) containing 10% FBS (Gibco, USA) and 1% P/S 100X (Bylabs, Hanam, Gyeonggi-do, Republic of Korea) and filtered via a 40-µm cell strainer to remove residual tissue fragments. The filtered cells were then cultured at 37 °C. After 24 h, non-adherent cells and other debris were removed by rinsing carefully with PBS and adding the fresh media. The adherent cells exhibiting a spindle-shaped morphology characteristic of dermal fibroblasts were cultured upto 90% confluence and expanded. Dermal fibroblasts from passage 3 to 4 were used in the experiments.

For the treatment, dermal fibroblasts were cultured into a 96-well plate at a density of 5 × 10^3^ cells/well. The cells were then treated with either CEXO or REXO at concentrations of 25, 50 or 100 µg/mL for 24 h at 37 °C. Cells that were untreated served as the control. The next day, cell viability was evaluated using a live/dead staining assay with acridine orange (AO) and propidium iodide (PI) (Sigma-Aldrich, MA, USA), and the cell proliferation rate was evaluated after trypsinization and counting. Moreover, the dermal fibroblasts were cultured with or without exosome formulations in a 6-well plate for 24 h and collected for RNA extraction. The quantitative reverse transcription polymerase chain reaction (qRT-PCR) was used to analyze the gene expression of the treated cells.

### Cellular uptake study of exosomes

To assess the cellular internalization of exosomes, both CEXO and REXO were labeled with the fluorescent lipophilic dye DiI (1,1'-Dioctadecyl-3,3,3',3'-Tetramethylindocarbocyanine Perchlorate, Invitrogen, USA) following the manufacturer's protocol. Briefly, exosomes were incubated with 2 µM of DiI at 37 °C for 30 min in the dark. Then, free dye was removed by using exosome spin columns (MW3000, Invitrogen, USA), followed by washing with PBS to eliminate residual unbound dye.

For the uptake assay, 5 X 10^4^ dermal fibroblast cells, isolated from dorsal skin of C57BL/6 mice, were seeded into 24-well plates and cultured overnight. The DiI-labeled exosomes (100 µg/mL) were added to the cells and incubated for 24 h at 37 °C in a humidified 5% CO₂ atmosphere. After incubation, cells were washed with PBS, stained with CellTracker™ Green CMFDA (Thermo Fisher, USA) and Hoechst 33342 (Thermo Fisher, USA) for cytoplasmic and nuclear visualization, respectively, and imaged using a fluorescence microscope (Nikon Instruments Inc., Japan). ImageJ software was used to quantify the exosome uptake per cell by analyzing DiI signal intensity.

### Induction of the hair loss model and exosome treatment

All animal-related procedures were conducted as per the standards certified from the Institutional Animal Care and Use Committee (IACUC)/Ethics Committee of Sungkyunkwan University (Suwon-si, Gyeonggi-do, Republic of Korea) and executed following the protocols for the treatment and management of laboratory animals. Approximately 2 × 3 cm² of the dorsal part of 7-week-old C57BL/6 mice was shaved and depilated with hair removal cream. The mice were left undisturbed for 3days to allow the skin to recover. After 3 days, CEXO or REXO with a dose equivalent to 100 µg exosomal proteins were administered at five points of the shaved area. The mice injected with PBS were considered as the control group. Hair regrowth was observed daily for 15 days and the percentage of hair growth was assessed by using ImageJ software. Then, following euthanization of mice, skin samples were taken for histological evaluation and for the assessment of gene expression profiles by qRT-PCR and protein expression by western blot analysis.

### qRT-PCR analysis

Total RNA was extracted from exosomes, exosomes treated dermal fibroblasts and skin samples using TRIzol (Thermo Fisher Scientific, USA). Skinsamples were lysed with the aid of a mechanical homogenizer (Taitec bead crusher homogenizer, Japan). cDNA synthesis was performed with the cDNA synthesis kit (Revertaid first strand, Thermo Fisher Scientific, USA) according to the manufacturer's instructions in the thermal cycler (Biometra TOne, Analytik Jena, Germany). qRT-PCR was conducted using Power SYBR Green PCR Master Mix (Thermo Scientific, USA) in the QuantStudio 6 flex real-time PCR system (Applied Biosystems', Thermo Scientific, USA). Relative gene expressions of each gene were calculated using comparative threshold (Ct) method and *GAPDH* as a reference gene. The sequences of the primers are presented in Table 1.

### Western blot analysis of skin tissue

Skin tissue samples were collected from mice 15 days after intradermal administration of CEXO, REXO, or PBS (control). The skin samples were homogenized in ice-cold RIPA buffer (Thermo Fisher Scientific, USA) supplemented with protease inhibitors (Thermo Fisher Scientific, USA). Protein concentrations were determined using the Pierce BCA Protein Assay Kit (Thermo Fisher Scientific, USA). Equal amounts of protein (20 µg) were loaded onto 12% SDS-PAGE gels and transferred to PVDF membranes (Millipore, USA). Membranes were blocked with 5% BSA in tris-buffered saline with 0.1% Tween 20 (TBST) for 1 h at room temperature and then incubated overnight at 4 °C with primary antibodies against Wnt-5a, Wnt-1a, β-catenin, Beclin-1, LC3B, PDGF-B (1:1000, Santa Cruz Biotechnology, USA), LC3A (1:1000, GeneTex, USA), VEGF-A (1:1000, BioLegend, USA), and GAPDH (1:50000, ABclonal, USA). Following primary antibody incubation, membranes were washed three times with TBST and incubated for 1 hour at room temperature with the appropriate HRP-conjugated secondary antibodies: goat anti-rabbit IgG (H + L) HRP (1:10000; Invitrogen, USA) for GAPDH and goat anti-rat IgG (H + L)-HRP (1:10000; Invitrogen, USA) for VEGF-A, and goat anti-mouse IgG (H + L)-HRP (1:10000; Invitrogen, USA) for the remaining markers. Protein bands were visualized using SuperSignal West Pico Chemiluminescent Substrate (Thermo Fisher Scientific, USA) in Fujifilm LAS-3000 Imager (Fujifilm, Tokyo, Japan). Band intensities were quantified using GelQuant.NET software, and protein expressions were normalized with GAPDH and expressed relative to the control group.

### Haematoxylin & Eosin (H&E) staining of the skin

Skin samples were fixed using 4% PFA solution for 24 h at RT, and subsequently paraffin embedding was done. The samples were then trimmed into 5 µm thick sections, followed by deparaffinization with xylene, rehydration through ethanol series, and staining with haematoxylin and eosin. Briefly, the sectioned tissues were treated with hematoxylin for 8 min, rinsed with water, followed by acid alcohol for 30 s, and dipped in eosin for 30 s. Then, the sections were dehydrated, rinsed in xylene, and fixed with coverslips. For morphological analysis, images of transverse and longitudinal sections were taken. The size and number of hair follicles were quantified from the stained sections.

### Statistical analysis

GraphPad Prism version 8.4.2 (GraphPad Software, Inc., USA) was utilized to create the graphs and conduct statistical interpretation. Unpaired two-tailed t-test, one-way ANOVA or two-way ANOVA with multiple comparisons tests were applied, as appropriate. A *p* value < 0.05 was considered statistically significant.

## Results

### Isolation and characterization of mouse adipose MSCs

**Figure 2:**
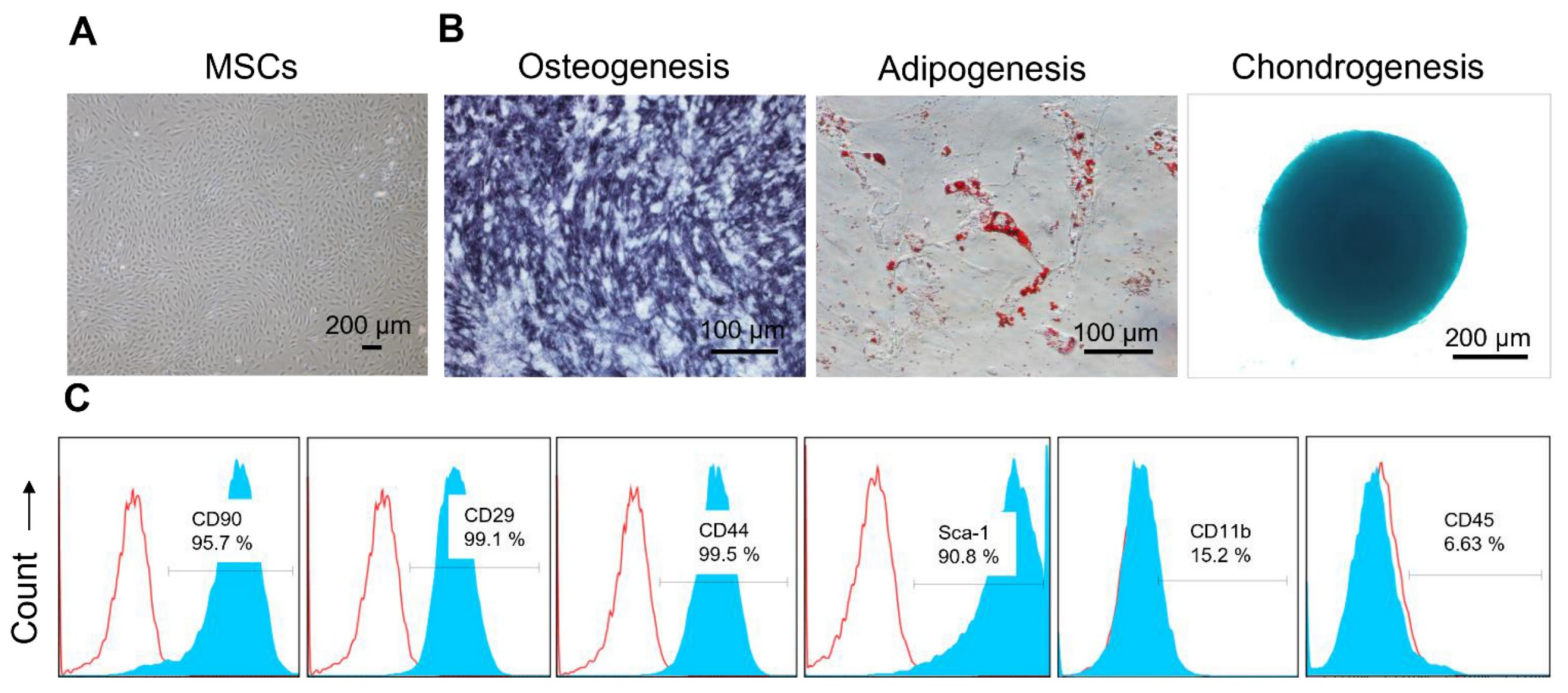
** Characterization of MSCs isolated from mouse adipose tissues.** (A) Representative bright-field image showing the morphology of MSCs isolated from mouse adipose tissue. (B) Representative images showing MSCs differentiated into osteocytes, adipocytes, and chondrocytes, as evidenced by Alkaline Phosphatase, Oil Red O, and Alcian blue staining, respectively. (C) Surface marker profiling of MSCs by flow cytometry. MSCs showed higher levels of CD90, CD29, CD44, and Sca-1, and lower levels of CD11b and CD45. The blue and red curves indicate samples stained with target antibodies and isotype control antibodies, respectively.

MSCs were successfully isolated from the subcutaneous adipose tissues of 8-week-old C57BL/6 mice and characterized by differentiation assays and flow cytometric analysis for surface markers. The isolated MSCs displayed a characteristic fibroblast-like appearance (Figure 2A) and demonstrated their ability to differentiate into various lineages. Specifically, osteogenic differentiation was evidenced by the dark blue staining in MSCs, indicating the presence of alkaline phosphatase activity; adipogenic differentiation by reddish lipid vesicles stained with Oil red O, appearing as red globules within the cells; and chondrogenic differentiation by Alcian blue-stained glycosaminoglycans (Figure 2B). In addition, these MSCs exhibited high expression of MSC-specific markers, including CD90, CD44, CD29, and Sca-1, while showing minimal expression of CD11b and CD45 (Figure 2C).

### Isolation and characterization of the exosomes

**Figure 3:**
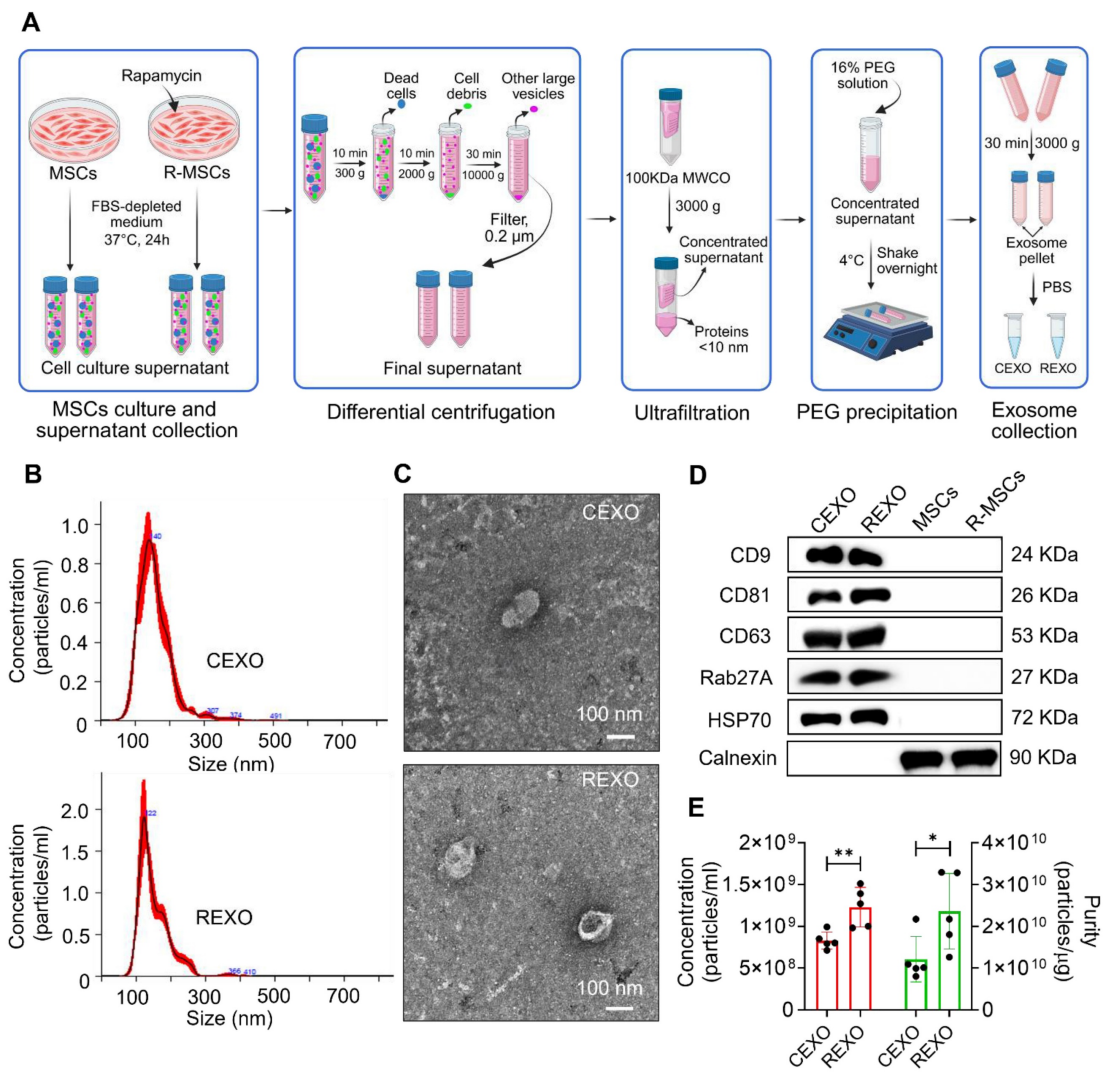
** Isolation and characterization of exosomes derived from control MSCs (CEXO) and exosomes derived from rapamycin-primed MSCs (REXO).** (A) Schematic illustration of the procedure followed for isolating the exosomes, created by biorender.com (B) Size distribution of EXO as measured by NTA. (C) Morphology of exosomes according to TEM imaging (scale bar, 100 nm). (D) Assessment of exosomal surface markers based on western blot analysis. (E) Purity of exosomes determined by NTA. Data are expressed as mean ± SD (n = 5) and were analyzed using unpaired two-tailed t-test. *p < 0.05, ** *p* < 0.01.

Exosomes were successfully isolated from MSCs and rapamycin-primed MSCs through a series of steps, involving differential centrifugation, ultrafiltration, and PEG precipitation, as illustrated in Figure 3A. NTA showed the average size of CEXO and REXO as 136.1 ± 9.8 nm, with a concentration of 8.50 × 10^8^ ± 4.12 × 10^6^ particles/mL, and 122.7 ± 4.1 nm, with a concentration of 1.27 × 10^9^ ± 6.74 × 10^7^ particles/mL, respectively (Figure 3B). The size distribution indicated a homogenous population of typically sized exosomes, with minimal contamination from larger vesicles or cellular debris, confirming the purity of the preparation. The characteristic morphology of CEXO and REXO was confirmed using TEM (Figure 3C). Western blot analysis demonstrated the detection of the exosome surface markers CD9, CD63, CD81, Rab27A, and HSP70. Calnexin, a cellular marker, was detected in MSCs and rapamycin-primed MSCs (R-MSCs) but was absent in both CEXO and REXO, confirming the purity of the isolated exosomes (Figure 3D). Based on NTA, the purity of the CEXO and REXO exosomes was found to be 1.43 × 10^10^ particles/µg and 2.79 × 10^10^ particles/µg, respectively (Figure 3E), indicating a high level of exosomal enrichment with minimal protein contamination.

### EXO treatment increases dermal cell proliferation and expression of genes related to Wnt/β-catenin signaling, autophagy, and growth factors

**Figure 4:**
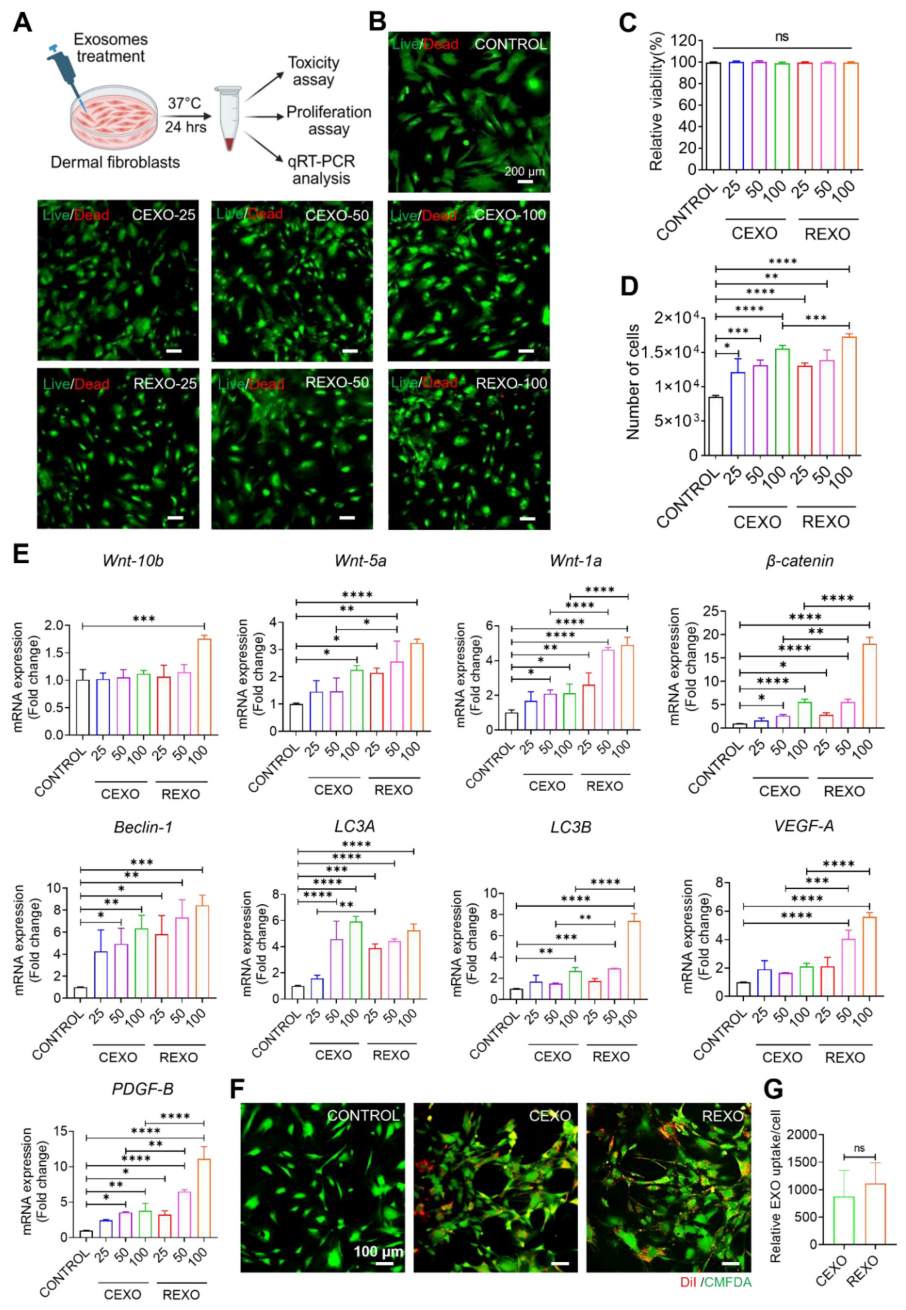
***In vitro* effect of exosomes on dermal fibroblasts.** (A) Illustration for treatment of exosomes for different analysis. Assessment of dermal cell viability after treatment with exosomes as determined by (B) live/dead staining assay, scale bars = 200 µm and (C) CCK-8 assay. (D) Assessment of dermal cell proliferation in response to exosome treatment, with results expressed as cell numbers compared to control. (E) Gene expression levels as evaluated by qRT-PCR, showing the gene expression levels linked to Wnt/β-catenin signaling (*Wnt10b, Wnt5a, Wnt1a, and β-catenin*), autophagy (*Beclin-1, LC3A, and LC3B*), and growth factors (*VEGF-A and PDGF-B*) in dermal fibroblasts treated with exosomes. (F) Representative images of the DiI-labeled CEXO and REXO into dermal fibroblasts (Red: DiI labeled EXOs, Green: Celltracker Green CMFDA). Scale bar = 100 µm (G) Quantification of exosomes uptake by dermal fibroblasts. Data are expressed as mean ± SD (n = 3) and were analyzed using an unpaired two-tailed t-test for comparing two groups, and one-way ANOVA with Tukey's multiple comparisons test for comparing more than 3 groups. * *p* < 0.05, ** *p* < 0.01, *** *p* < 0.001, ****p < 0.0001, and ns = not significant.

Previous studies indicated that exosomes derived from MSCs contain bioactive compounds, including proteins, lipids, and RNAs that can regulate cellular processes by affecting the survival, proliferation, and gene expression of recipient cells 42-44. To assess the *in vitro* effects of CEXO and REXO on dermal fibroblasts, several doses of exosomes were treated to the cells, and were then evaluated in terms of cell viability, proliferation, and gene expression (Figure 4A). No cytotoxicity was observed at any CEXO or REXO dose (25, 50, or 100 µg/mL), as confirmed by both AO/PI staining and CCK-8 assay (Figure 4B and 4C), in agreement with previous reports showing that MSC-derived exosomes are non-toxic and promote cell survival 45, 46.

In the proliferation assay, both CEXO and REXO treatments caused a dose-dependent rise in dermal cell numbers, with the highest dose (100 µg/mL) of CEXO and REXO causing the greatest increase in cell counts (Figure 4D). This increase in proliferation is consistent with studies demonstrating MSC-EXO-induced cell proliferation in various tissue types 29, 47-51. However, the enhanced proliferation observed with REXO compared to that with CEXO suggests that rapamycin priming might have further augmented the regenerative potential of exosomes 32, 52.

Similarly, gene expression analysis of EXO-treated dermal fibroblasts using qRT-PCR revealed significant upregulation of Wnt/β-catenin signaling genes (*Wnt10b, Wnt5a, Wnt1a, β-catenin*), autophagy-related genes (*Beclin-1, LC3A, LC3B*), and growth factors (*VEGF-A and PDGF-B*) in a dose-responsive manner following exosome treatment, with REXO showing greater activation than CEXO (Figure 4E). Most genes showed the highest expression levels at 100 µg/mL (*p* < 0.001). This enhanced gene expression aligns with the findings of previous research studies demonstrating the function of MSC-derived exosomes in promoting Wnt/β-catenin signaling and autophagy, both of which are critical for dermal cell proliferation and hair follicle growth 53, 54. The superior gene activation observed in the REXO-treated cells supports that rapamycin priming enhances the biological activity of MSC exosomes, potentially through the promotion of autophagy pathways, which is in line with earlier findings, showing that rapamycin improves cellular responses to exosome therapy 55, 56.

To evaluate the internalization of exosomes in dermal fibroblast cells, we treated cells with 100 µg/mL of DiI-labeled CEXO or REXO. Representative fluorescence images demonstrated the intracellular localization of DiI-labeled exosomes by dermal fibroblasts, as evidenced by red fluorescence (DiI) localized within the green-labeled cytoplasm (CellTracker Green) (Figure 4F). Quantitative analysis of exosome uptake per cell by ImageJ revealed no significant difference in exosome uptake between the two groups, as measured by comparable mean DiI signal intensities per cell (Figure 4G).

### REXO enhances hair regrowth in mice

**Figure 5:**
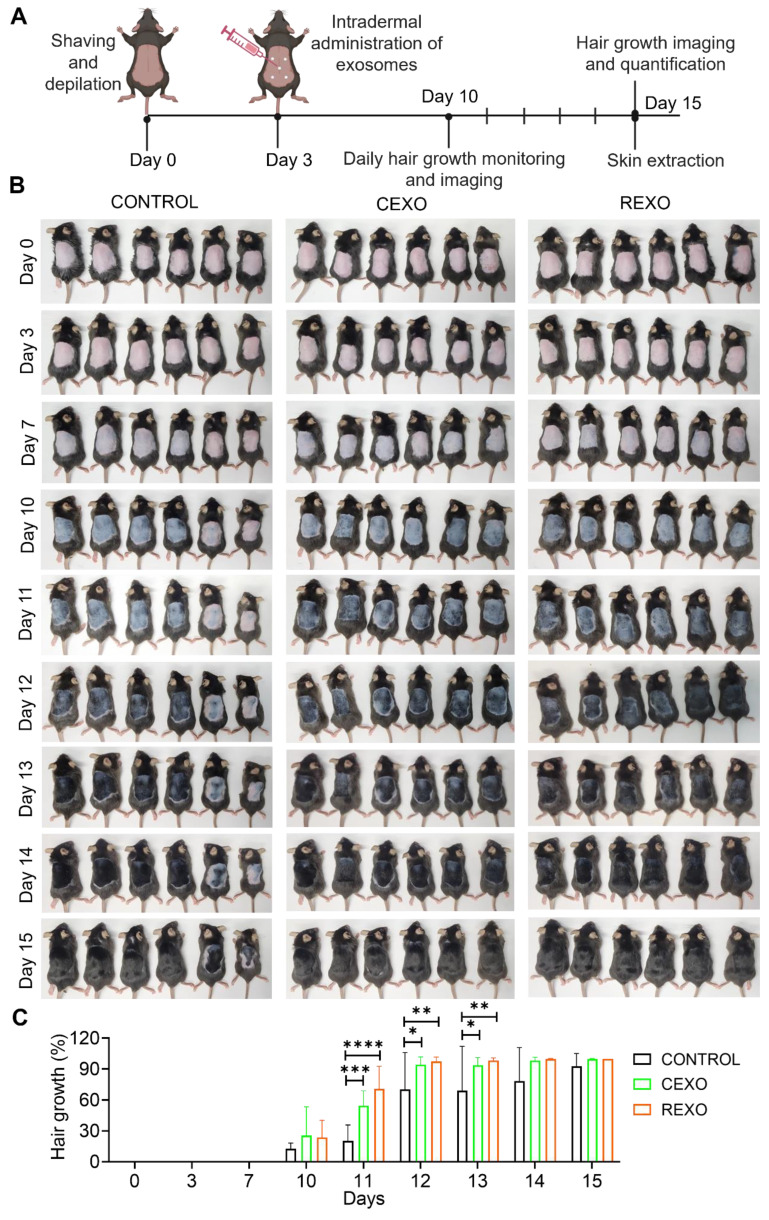
** Enhanced hair growth induced by rapamycin-primed MSC-derived exosomes (REXO) in an *in vivo* mouse model**. (A) Schematic of the hair growth model timeline, showing the key time points for exosome administration and evaluation. (B) Images of mice on days 0, 3, 7, 10, 11,12, 13, 14 and 15 post-exosome treatment, illustrating hair growth progression (n = 6). (C) Percentage of hair growth in mice (as determined by ImageJ). Data are represented as mean ± SD (n = 6 per group) and were analyzed by two-way ANOVA followed by Tukey's multiple comparisons test. * *p* < 0.05, ** *p* < 0.01, *** *p* < 0.001 and **** *p* < 0.0001.

To evaluate the hair growth potential of exosomes derived from rapamycin-primed MSCs (REXO), approximately 2 × 3 cm^2^ of the dorsal part of C57BL/6 mice (n = 6 per group) were shaved and depilated with hair removal cream. The mice were left undisturbed for 3 days to allow the skin to recover and synchronize hair follicles in the telogen phase prior to treatment. On day 3, we treated the mice with CEXO, REXO, or with PBS as the untreated control group. Hair regrowth was visually monitored across the treatment groups over a predetermined period (Figure 5A). The in vivo hair regrowth outcomes of REXO treatment were compared with those of CEXO and an untreated control group. Specifically, 100 µg of exosomes were administered at five evenly distributed points on the shaved area on Day 0. By Day 10, both REXO and CEXO treatments had resulted in diffuse darkening of the dorsal skin, indicative of hair follicles entering the anagen growth phase of the hair growth cycle (Figure 5B). Some untreated control mice also showed mild skin darkening, though with greater individual variability in response. By Day 15, images of the dorsal skin revealed that mice in the REXO group exhibited almost complete hair regrowth, with consistent hair density observed across all mice in that group. In contrast, the CEXO and untreated control groups displayed variable outcomes, with some mice showing minimal regrowth and others exhibiting greater coverage, although none matched the uniformity observed in the REXO-treated group (Figure 5B). The hair growth percentage as calculated by ImageJ showed significant increased hair growth in REXO-treated mice by day 11 compared to CEXO and CONTROL groups (Figure 5C). This trend continued to day 12 and day 13, with REXO-treated mice showing superior hair growth. By day 14, hair growth reached a plateau across all groups, with no further significant differences observed between treatments. These results indicate that REXO accelerates hair growth more effectively than CEXO, particularly during the early active growth phase of the observation period. On day 15, after imaging, the mice were euthanized, and skin were extracted for histological study and PCR analysis for cytokines.

### REXO modulates Wnt signaling, autophagy and growth factor expression

**Figure 6:**
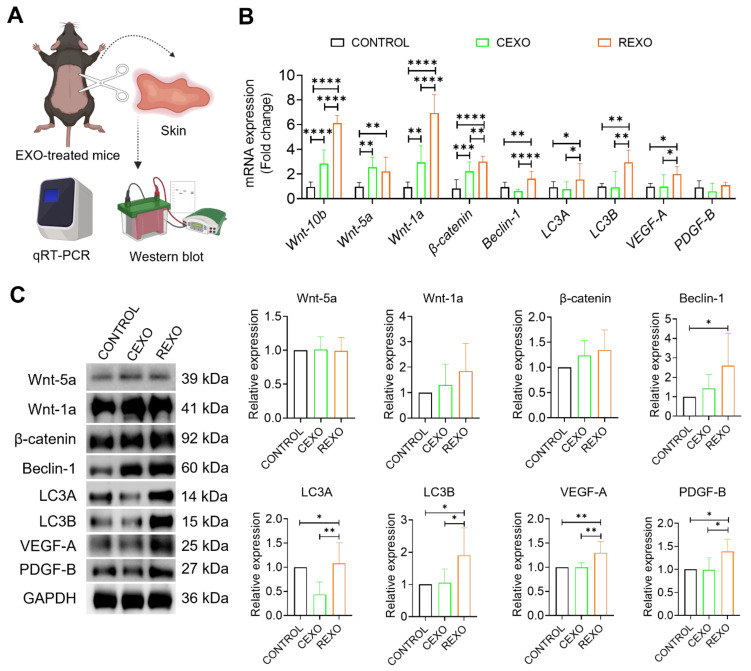
** REXO enhances hair regrowth by promoting Wnt signaling, autophagy, and growth factors expression.** (A) Schematic overview of *in vivo* sample processing and analysis. (B) Relative mRNA expression levels of key genes associated with Wnt/β-catenin signaling (*Wnt-10b, Wnt-5a, Wnt-1a, and β-catenin*), autophagy (*Beclin-1, LC3A, and LC3B*), and growth factors (*VEGF-A and PDGF-B*) in skin samples extracted from PBS-treated CONTROL, CEXO, and REXO treated mice. (C) Relative protein expressions of the same targets analyzed by western blot. Data are expressed as mean ± SD (n = 6 per group) and were analyzed using one-way ANOVA with Tukey's multiple comparisons test. * *p* < 0.05, ** *p* < 0.01, ***p < 0.001, and ****p < 0.0001.

To assess the biological effects of EXO treatment in vivo, we analyzed skin tissues collected 15 days after intradermal administration of CEXO, REXO or PBS (CONTROL). Gene expressions were evaluated by qRT-PCR and protein expressions by western blot. qRT-PCR revealed that REXO treatment significantly upregulated genes linked to the Wnt/β-catenin signaling cascade, autophagy, and growth factor expression in the skin of C57BL/6 mice (Figure 6A). Specifically, REXO-treated mice exhibited increased mRNA levels of Wnt signaling genes, including *Wnt10b (*6.13 ± 0.54-fold,* p* < 0.0001)*, Wnt-5a (*2.21 ± 0.85-fold, *p* < 0.01)*, Wnt-1a (*6.93 ± 1.36-fold, *p* < 0.0001)*,* and *β-catenin (*3.01 ± 0.37-fold, *p* < 0.0001), in comparison with the CONTROL group. In addition, REXO treatment resulted in increased expression of autophagy-related genes *Beclin-1 (*1.62 ± 0.52-fold, *p* < 0.01)*, LC3A (*1.55 ± 1.25-fold, *p* < 0.05)*, and LC3B (*2.95 ± 0.93-fold, *p* < 0.01), indicating that REXO promotes autophagy. Growth factors, such as *VEGF-A (*1.99 ± 0.44-fold, *p* < 0.05)* and PDGF-B (*1.60 ± 0.29-fold), which are critical for hair follicle development, also demonstrated higher expression in the REXO group than in the CEXO or CONTROL groups, indicating enhanced regenerative and hair growth-promoting effects.

To determine whether the observed changes in mRNA expression were reflected at the protein level, we performed western blot analysis of skin tissue samples. Consistent with the gene expression analysis, western blot analysis also revealed increased expression of several proteins associated with hair growth, autophagy and growth factors. REXO treated mice skin samples demonstrated significant increase in expression of beclin-1, LC3A, LC3B, VEGF-A and PDGF-B compared to both CEXO and CONTROL groups (Figure 6B). Wnt-1a and β-catenin also showed higher expression in REXO than in CEXO treated mice although these increases were not statistically significant. Interestingly, LC3A expression was significantly decreased in the CEXO-treated group (*p* < 0.01) compared to CONTROL, while it was significantly increased in the REXO-treated group (*p* < 0.01) compared to CEXO-treated group. Collectively, these results support the hypothesis that REXO promotes hair follicle regeneration by upregulating key proteins involved in Wnt signaling, autophagy, and growth factor pathways, thereby reinforcing the mRNA-level findings and demonstrating coordinated molecular changes induced by rapamycin-primed MSC-derived exosome treatment.

To further investigate the potential mechanisms by which REXO influences hair growth, we conducted qRT-PCR analysis of exosomes themselves. REXO demonstrated a significant upregulation of mRNAs for *Wnt-1a* (3.32 ± 0.05-fold, *p* < 0.0001), *Beclin-1* (1.58 ± 0.04-fold, *p* < 0.01), *LC3A* (1.24 ± 0.04, *p* < 0.05), *LC3B* (1.51 ± 0.53, *p* < 0.05), *VEGF-A* (1.56 ± 0.13-fold, *p* < 0.05), and *PDGF-B* (2.57 ± 0.12-fold, *p* < 0.001) in REXO relative to CEXO (Figure S1). Modest increases were noted for *Wnt-5a and β-catenin* transcripts, while* Wnt-10b* was undetectable in either exosome. These findings indicate that rapamycin priming of MSCs leads to the selective enrichment of exosomal transcripts encoding for crucial signaling molecules and growth factors implicated in hair follicle development, maintenance, and supporting vascularization, potentially contributing to the augmented hair-growth promoting effects observed with REXO treatment.

### Histological analysis of skin samples after exosome treatment

**Figure 7:**
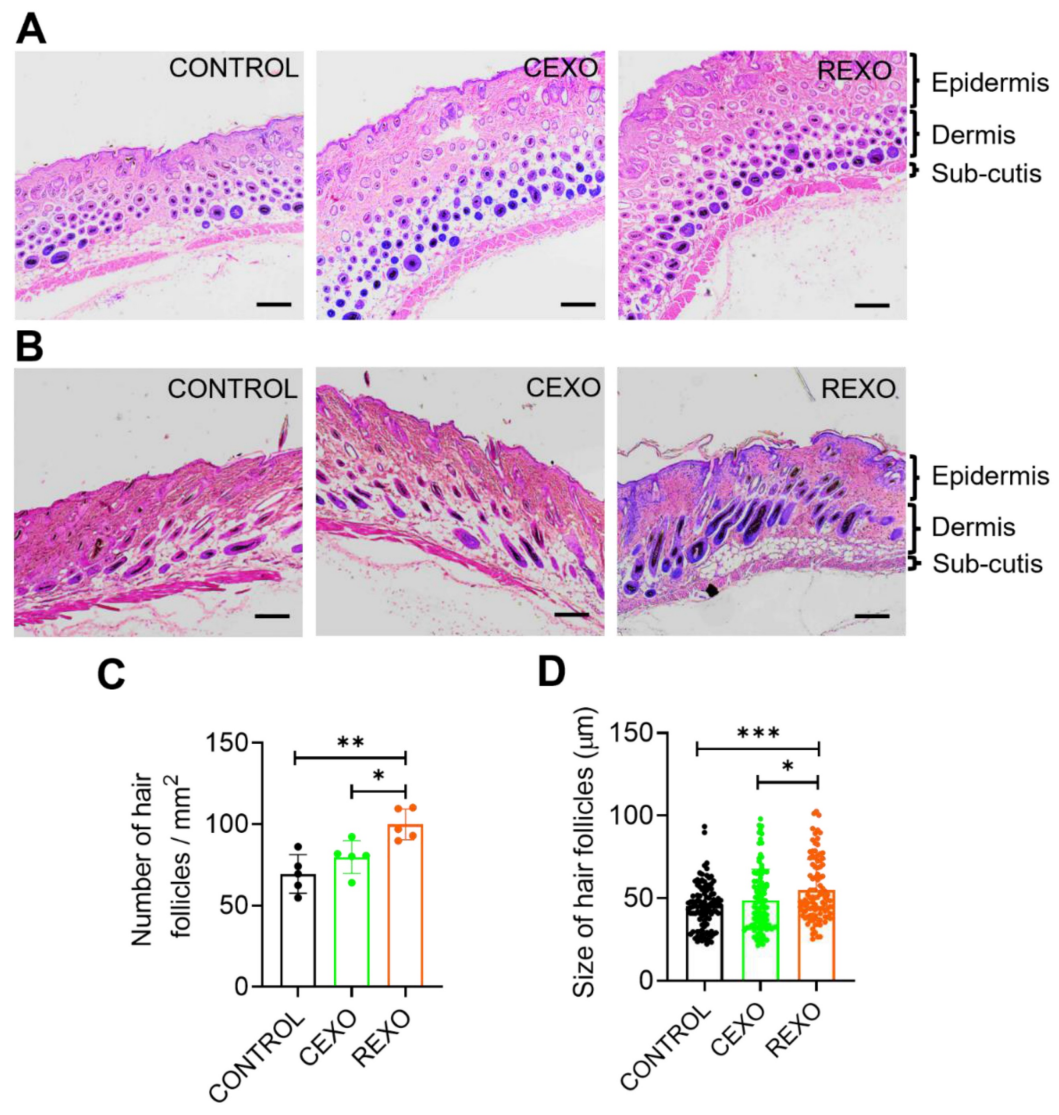
**REXO promotes the anagen phase in the hair growth cycle.** Representative hematoxylin and eosin (H & E) stained images of skin sections from the mice treated with exosomes showing (A) transverse and (B) longitudinal views. Scale bar = 200 µm. (C) Quantification of number of hair follicles per area. (D) Size distribution of hair follicles. Data are expressed as mean ± SD (n = 5 per group) and were analyzed using one-way ANOVA with Tukey's multiple comparisons test. * *p* < 0.05, ** *p* < 0.01 and ***p < 0.001.

On Day 15, histological analysis of H&E-stained skin sections from the REXO, CEXO, and CONTROL groups was conducted, including both transverse (Figure 7A) and longitudinal sections (Figure 7B), to evaluate hair follicle structure, number, and size. In the REXO-treated group, transverse and longitudinal sections revealed a notably higher density of hair follicles compared with those noted to the CEXO and CONTROL groups, with a larger proportion of active follicles in the anagen phase. Representative images of sections stained with H&E from the REXO group showed a significantly more uniform distribution and greater number of well-defined, large, elongated hair follicles extending deep into the dermal layer, indicating robust follicular development and active hair growth. In contrast, the CEXO-treated and CONTROL samples displayed predominantly round, fewer, smaller follicles, with dark, dense centers indicative of the telogen phase or the early stages of the hair growth cycle. Quantitative analysis confirmed that the REXO-treated mice had a significantly higher number of hair follicles per area (Figure 7C) and greater average hair follicle size (Figure 7D) than CEXO-treated and CONTROL mice, underscoring the superior regenerative potential of exosomes from rapamycin-primed MSCs in enhancing hair follicular development and hair growth in this model.

## Discussion

The objective of this study was to meet the growing demand for effective hair loss treatments by investigating an innovative approach that utilizes exosomes from rapamycin-primed mesenchymal stem cells (MSCs) to stimulate hair regrowth. Hair loss remains a widespread concern, making it essential to introduce new and innovative therapies that provide lasting hair regrowth with minimal side effects 57. Currently available therapies for hair loss are limited by their efficacy, side effect profile, cost, and tolerability 6. Although stem cell-based therapies have shown promise, they come with challenges such as immune rejection, tumorigenicity, and complex handling requirements 7, which have prompted researchers to explore paracrine factors, such as extracellular vesicles (EVs) and exosomes, as alternative treatments 58, 59. Exosomes are paracrine agents derived from MSCs and offer a promising alternative due to their stability, ease of administration, and potent bioactivity 60, 61. Previous studies showed that exosomes from MSCs demonstrated regenerative potential in various tissues 60, 62, while priming MSCs with rapamycin have been shown to modulate MSCs functions favorably in several contexts, including immunomodulation and anti-inflammatory effects 32, 63, 64. However, the isolation and impact of exosomes derived from rapamycin-primed MSCs, specifically for hair growth, has not yet been reported. Therefore, the present study hypothesized that rapamycin-primed MSCs could yield exosomes with enhanced hair growth-promoting effects.

In this research study, MSCs were successfully isolated from mouse adipose tissue and exosomes from adipose tissue-derived MSCs (CEXO) and rapamycin-primed MSCs (REXO). The combination of differential centrifugation, ultrafiltration, and PEG precipitation offers a streamlined approach for high-purity exosome isolation. Differential centrifugation effectively removes cellular debris and larger particles, yielding an initially purified exosome sample 65, 66. Ultrafiltration further enhances purity by concentrating exosomes and filtering out smaller protein contaminants 67, while PEG precipitation efficiently aggregates and isolates exosomes without compromising their structural integrity 68, 69. Additionally, this precipitation-based method is simple, easy, inexpensive, rapid, and does not require any specific equipment 70, 71. The isolated exosomes aligned with the shape and size of exosomes reported in previous studies 72, 73. CEXO and REXO expressed specific exosomal markers, namely CD9, CD81, CD63, Rab27A, and HSP70 while calnexin was not detected, confirming that the isolation was pure, without contaminating cell organelles and apoptotic bodies 74, 75. The high particle-to-protein ratio observed in the NTA analysis confirmed the purity of the exosomes, as reported in previous research studies 76, 77, indicating minimal protein contamination and ensuring the high quality of exosomes for downstream applications.

The viability and proliferation of dermal fibroblasts are crucial for hair growth, as they exhibit a significant role in maintaining follicular health and enhancing the shift of hair follicles from the telogen to the anagen phase, ultimately supporting overall hair regeneration 78, 79. Our results showed that REXO had a significant effect on dermal fibroblasts, showing no cytotoxicity even at higher doses and promoting greater cell proliferation compared to CEXO. Moreover, the uptake of exosomes by dermal fibroblasts was successfully visualized using fluorescence microscopy, confirming the internalization of DiI-labeled exosomes. However, the quantitative analysis revealed no significant difference in uptake efficiency between REXO and CEXO. This suggests that the enhanced biological effects of REXO are more likely attributed to differences in exosomal cargo composition rather than variations in uptake dynamics. This further supports the notion that rapamycin priming modulates the functional payload of MSC-derived exosomes, contributing to their superior regenerative potential.

The findings of the present study support previous research, which has highlighted MSC-derived exosomes as potent agents for tissue regeneration and hair growth, particularly through their influence on Wnt/β-catenin signaling, autophagy, and the secretion of growth factors 80, 81. The Wnt/β-catenin cascade is crucial for regulating hair follicle cycling and activation of dermal papilla cells 82, with studies showing that Wnt activation is necessary for the induction of the anagen phase in hair follicles 83, 84. qRT-PCR analysis conducted on dermal fibroblasts treated with exosomes, as well as on skin samples obtained from exosome-treated mice, showed that REXO treatment upregulated Wnt-associated genes, such as *Wnt10b, Wnt5a, Wnt1a, and β-catenin* more effectively than CEXO, suggesting rapamycin priming may enhance the exosomal transfer of Wnt ligands or signaling mediators, or modulate the signaling activity in recipient cells, resulting in a stronger activation of hair follicle stem cells and anagen induction.

The effect of rapamycin on autophagy regulation is well-documented, as the inhibition of mTOR signaling promotes autophagy, allowing for cellular maintenance and repair that supports tissue regeneration 85-87. Sun *et al*. (2024) demonstrated that autophagy is essential for hair follicle stem cell activation and regeneration, as it regulates glycolysis and promotes enhanced hair growth 36. In our study, REXO-treated dermal fibroblasts and REXO-treated mice skin exhibited increased expression of autophagy-associated genes, such as Beclin-1, LC3A, and LC3B, indicating enhanced autophagic activity. This is likely due to rapamycin's effects on MSCs, which prime exosomes with autophagy-promoting factors. These data suggest that REXO may have improved the autophagic activity of recipient cells, thereby enabling efficient cellular recycling and reducing cellular stress, both of which are beneficial for follicular activity and hair regeneration. Future studies should therefore conduct a detailed molecular pathway analysis of REXO activation and examine the specific contents of REXO cargo to identify factors that may enhance autophagy and support hair follicle activity.

In addition, the increased expression of growth factors, such as VEGF-A and PDGF-B, in the REXO-treated mice skin samples reflected enhanced follicle vascularization and nutrient supply. Our results also revealed that VEGF-A and PDGF-B gene expressions were significantly increased with exosome treatment in a dose-dependent manner. Growth factors are essential for supporting hair follicle health 88, 89, and their increased expression in the REXO group further validated the regenerative advantage of rapamycin priming in MSC exosome-based therapies. Other studies have similarly found that MSC-derived exosomes enriched the production of growth factors, which in turn can improve vascularization and nutrient support in scalp tissues, providing the necessary support for hair follicle maintenance and growth 90. In this study, we used C57BL/6 mice to examine the *in vivo* effects of REXO on hair regrowth, and we showed that the intradermal administration of REXO promoted more uniform and robust hair regrowth in treated mice by day 15 post-treatment, indicating a transition to the growth phase of hair cycle. qRT-PCR and western blot analysis of skin samples further provided insights into the differential effects of REXO and CEXO on key genes and proteins involved in hair growth and autophagy. Our findings suggest that REXO modulates Wnt signaling at multiple levels. The observed trend towards increased expression of Wnt and autophagy- related genes and proteins in REXO-treated samples suggests that REXO promoted Wnt signaling, a pathway known to be crucial for hair follicle development and regeneration 24, 91. Although Wnt-1a and β-catenin protein levels also appeared elevated in REXO-treated samples, these changes did not reach statistical significance, possibly due to post-transcriptional regulation, differences in protein turnover, or the modest magnitude of biological effect 92, 93. Interestingly, while Wnt-5a mRNA was significantly upregulated in both CEXO and REXO groups, this increase was not reflected at the protein level, suggesting post-transcriptional modulation. Moreover, as Wnt-5a is a component of the non-canonical Wnt pathway, it is subject to distinct regulatory mechanisms compared to canonical Wnt components like Wnt-1a 94, 95. These findings suggest that REXO promotes hair growth by predominantly influencing canonical Wnt signaling via Wnt-1a, suggesting a limited role for non-canonical Wnt signaling 82. Moreover, the trend towards increased PDGF-B and VEGF-A expression in REXO-treated samples indicates enhanced role in promoting hair follicle growth 96, 97. The significant decrease in LC3A expression in CEXO-treated protein samples, coupled with the significant increase in LC3A expression in REXO-treated samples, suggests differential effects on autophagy. The results indicate that REXO treatment enhanced autophagy, while CEXO treatment suppressed it, indicating that REXO was more effective in stimulating hair growth by modulating autophagic pathway 36, 98. Overall, these findings suggest that REXO modulates multiple regenerative pathways more effectively than CEXO, including canonical Wnt signaling, autophagy, and angiogenesis, potentially contributing to enhanced hair follicle activation promoting pathways associated with hair growth compared to CEXO. Histological analysis further revealed an increased number of large, elongated hair follicles extending deep into the dermis, suggesting active hair follicular development 99, 100.

Moreover, qRT-PCR analysis of exosomal mRNA revealed a selective modulation of hair growth-related factors in response to rapamycin priming of MSCs. Notably, *Wnt-1a* mRNA, a known promoter of hair follicle regeneration and dermal papilla cell activation 15, 101, was significantly upregulated in REXO. This suggests that rapamycin priming enhances the selective packaging of Wnt-1a into exosomes, indicating a mechanism by which cellular preconditioning can dynamically alter exosomal cargo to potentiate targeted therapeutic outcomes. In addition, REXO showed significantly elevated mRNA levels of *Beclin-1, LC3A and LC3B*, implicating an enhancement of exosome-associated autophagic signaling, which has been linked to the maintenance of hair follicle integrity and cycling 36. Furthermore, *VEGF-A*, and *PDGF-B,* which are crucial growth factors involved in hair follicle development and supporting vascularization 97, 102, were also significantly upregulated in REXO, indicating a multifaceted contribution of the modified exosomal cargo to the observed enhanced hair regrowth. The modest and non-significant rise in *Wnt-5a* and *β-catenin* mRNA in REXO suggests the possibility of post-transcriptional or translational regulation not fully captured at the transcriptomic level. While *Wnt-10b* mRNA remained undetected in both groups, it is possible that the protein itself may still be present at functional levels, emphasizing the need for proteomic validation. Overall, the selective enrichment of pro-anagenic transcripts—especially Wnt-1a, autophagy mediators, and angiogenic factors—in REXO underscores the potential of rapamycin priming as a strategy to tailor the therapeutic efficacy of MSC-derived exosomes. This finding supports a model in which synergistic effects of multiple signaling components mediate enhanced hair follicle activation and regeneration, highlighting the context-dependent and dynamic nature of exosomal cargo in regenerative medicine applications.

The present study explored the therapeutic efficacy of exosomes isolated from MSCs primed with rapamycin to enhance hair growth. Our aim was to develop a novel therapeutic approach for hair loss by exploring how rapamycin-primed MSC-derived exosomes affect hair growth. However, our study has some limitations. We used dermal fibroblasts, which, while relevant and widely accepted model for studying skin-hair follicle interactions 103, 104, do not fully represent the complex cellular composition of the hair follicle. While dermal papilla cells (DPCs) are central to hair follicle induction, our study focused on exosome-mediated effects on the dermal fibroblasts, which are key components of the dermal compartment and contribute to hair follicle support and regeneration 105, 106. Dermal fibroblasts are essential for hair follicle development and cycling, as they not only produce the extracellular matrix but also secrete key signaling molecules such as Wnt ligands, VEGF, and PDGF 107. These functions enable fibroblasts to support the hair follicle niche, influence dermal papilla-like activity, and facilitate cross-talk with hair follicle epithelial cells, thereby contributing to hair growth and regeneration 108, 109. While our results provide insights into exosome-mediated modulation of dermal fibroblasts, we acknowledge the need for future studies involving purified DPCs and other specialized follicular cell types. Further experiments such as study of REXO effect on activation of hair-inductive signaling pathways, and functional assays relevant to hair follicle initiation and cycling will be essential to fully elucidate the cell-type-specific mechanisms underlying exosome-mediated hair follicle regeneration. Next, we only conducted a morphological study, without exploring the in-depth mechanism dictating this process. Thus, further studies focusing on the underlying mechanism should be carried out in future. Moreover, the cargo of exosomes released by MSCs can undergo significant changes in response to rapamycin treatment, including alterations in miRNAs, proteins, lipids, mRNAs, lncRNAs, and other small non-coding RNAs. These changes can collectively determine the biological effects of exosomes on the recipient cells, potentially influencing their proliferation, survival, and other behaviors. While our study identified key mRNA transcripts within exosomes via qRT-PCR only, to provide deeper mechanistic understanding, comprehensive profiling such as RNA sequencing, proteomics, miRNA microarray analysis and lipidomics would be necessary to profile the exosome cargo after rapamycin treatment.

In conclusion, both *in vitro* and *in vivo* experiments in this research indicated that exosomes derived from rapamycin-primed MSCs have superior restorative effects on hair growth compared with naive MSC exosomes, highlighting the potential benefits of rapamycin priming in enhancing exosome-mediated therapeutic effects for hair regrowth. These findings provide a foundation for further exploration into exosome-based therapies for hair loss, making REXO as a promising candidate for clinical applications.

## Supplementary Material

Supplementary figure.

## Figures and Tables

**Table 1:** Primers used for qRT-PCR

Gene	Forward primer sequence	Reverse primer sequence
GAPDH	ACCACAGTCCATGCCATCAC	TCCACCACCCTGTTGCTGTA
Wnt-10b	TTCTCTCGGGATTTCTTGGATTC	TGCACTTCCGCTTCAGGTTTTC
Wnt-5a	CTCCTTCGCCCAGGTTGTTATAG	TGTCTTCGCACCTTCTCCAATG
Wnt-1a	CGAGAGTGCAAATGGCAATTCCG	GATGAACGCTGTTTCTCGGCAG
β-catenin	ATCCAAAGAGTAGCTGCAGG	TCATCCTGGCGATATCCAAG
Beclin-1	CAGCCTCTGAAACTGGACACGA	CTCTCCTGAGTTAGCCTCTTCC
LC3A	CTGCCTGTCCTGGATAAGACCA	CTGGTTGACCAGCAGGAAGAAG
LC3B	GACGGCTTCCTGTACATGGTTT	TGGAGTCTTACACAGCCATTGC
VEGF-A	CTGCTGTAACGATGAAGCCCTG	GCTGTAGGAAGCTCATCTCTCC
PDGF-B	AATGCTGAGCGACCACTCCATC	TCGGGTCATGTTCAAGTCCAGC

## Abbreviations

AO: acridine orange
BCA: bicinchoninic acid
CEXO: non-primed MSC-derived exosomes
DMEM: dulbecco's modified eagle medium
EVs: extracellular vesicles
EXO: exosome
FBS: fetal bovine serum
H&E: Haematoxylin & Eosin
IACUC: institutional animal care and use committee
MSC: mesenchymal stem cells
mTOR: mechanistic target of rapamycin
NTA: nanoparticle tracking analysis
PBS: phosphate-buffered saline
PDGF-B: platelet-derived growth factor b
PEG: poly(ethylene glycol)
PI: propidium iodide
P/S: penicillin/streptomycin
qRT-PCR: quantitative reverse transcriptase polymerase chain reaction
REXO: rapamycin-primed MSC-derived exosomes
R-MSCs: rapamycin-primed MSCs
RT: room temperature
TEM: transmission electron microscopy
VEGF-A: vascular endothelial growth factor a
