# Supplementary material for: Enhancing hair regrowth using rapamycin-primed mesenchymal stem cell-derived exosomes
Source: Theranostics. 2025 Jun 9;15(14):6938–56. doi: 10.7150/thno.107659 (PMC12203813; doi:10.7150/thno.107659)
Supplement: Supplementary file 1 — Supplementary figure. [file thnov15p6938s1.pdf]

Supplementary figure for

Enhancing hair regrowth using rapamycin-primed mesenchymal stem cell-derived exosomes

Manju Shrestha[a], Tiep Tien Nguyen[a], Rabyya Kausar[a], Dinesh Chaudhary[a], Ines Ferdiana Puspitasari[a], Hu-Lin Jiang[b,c,d,e*], Hyung-Sik Kim[f,g,h,*], Simmyung Yook [i,j,*] and Jee-Heon Jeong[a*]

[a]*Department of Precision Medicine, School of Medicine, Sungkyunkwan University, Suwon 16419, Republic of Korea*

[b]*State Key Laboratory of Natural Medicines, China Pharmaceutical University, Nanjing, 210009, China*

[c]*Jiangsu Key Laboratory of Druggability of Biopharmaceuticals, China Pharmaceutical University, Nanjing, 210009, China*

[d]*Jiangsu Key Laboratory of Drug Discovery for Metabolic Diseases, China Pharmaceutical University, Nanjing, 210009, China*

[e]*NMPA Key Laboratory for Research and Evaluation of Pharmaceutical Preparations and Excipients, China Pharmaceutical University, Nanjing, 210009, China*

[f]*Department of Oral Biochemistry, Dental and Life Science Institute, Pusan National University, Yangsan, 50612, Republic of Korea*

[g]*Department of Life Science in Dentistry, School of Dentistry, Pusan National University, Yangsan, 50612, Republic of Korea*

[h]*Education and Research Team for Life Science on Dentistry, Pusan National University, Yangsan, 50612, Republic of Korea*

[i]*School of Pharmacy, Sungkyunkwan University, Suwon, Gyeonggi, 16419, Republic of Korea*

[j]*Department of Biopharmaceutical Convergence, Sungkyunkwan University, Suwon, 16419, Republic of Korea*

*Corresponding authors

Jee-Heon Jeong–Department of Precision Medicine, School of Medicine, Sungkyunkwan University, Suwon, Gyeonggi 16419, Republic of Korea; Email: jeeheon@skku.edu, Tel: +82-31-

299-6165

Simmyung Yook–Department of Biopharmaceutical Convergence, Sungkyunkwan University, Suwon, Gyeonggi 16419, Republic of Korea; Email: ysimmyung@skku.edu; Tel: +82-31-290-7728

Hyung-Sik Kim–Department of Oral Biochemistry, Dental and Life Science Institute, Pusan National University, Yangsan 50612, Republic of Korea; Email: hskimcell@pusan.ac.kr; Tel: +82-51-510-8231

Hu-Lin Jiang– State Key Laboratory of Natural Medicines, China Pharmaceutical University

Nanjing 211198, China; E-mail: jianghulin3@gmail.com; Tel: +82-025-8327-1299

Supplementary figure

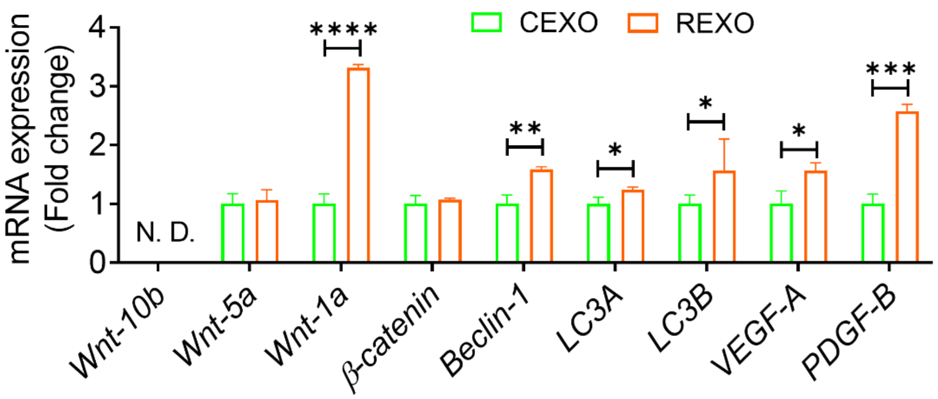

Figure S1. Effect of rapamycin priming on exosomal gene expression. Relative mRNA expression levels in exosomes derived from CEXO and REXO. Target genes include components of the key Wnt/β-catenin signaling (*Wnt-10b, Wnt-5a, Wnt-1a, and β-catenin*), autophagy (*Beclin-1, LC3A, and LC3B*), and growth factors (*VEGF-A and PDGF-B*) related genes in exosomes derived from control and rapamycin-primed MSCs. Data are presented as mean ± SD (n = 3) and were analyzed using unpaired two-tailed t-test. * $p < 0.05$, ** $p < 0.01$, ***$p < 0.001$, and ****$p < 0.0001$. N. D.= Not detected
